# AQP4-specific T cells determine lesion localization in the CNS in a model of NMOSD

**DOI:** 10.1186/s40478-025-01947-8

**Published:** 2025-02-11

**Authors:** Ali Maisam Afzali, Oleksii Ulianov, Luise Eckardt, Ingrid Stas, Lea Seeholzer, Katja Steiger, Doron Merkler, Thomas Korn

**Affiliations:** 1 Institute for Experimental Neuroimmunology, Technical University of Munich School of Medicine and Health, Ismaninger Str. 22, 81675 Munich, Germany; 2 Department of Neurology, Technical University of Munich School of Medicine, Ismaninger Str. 22, 81675 Munich, Germany; 3 Institute of Pathology, Technical University of Munich, Trogerstr. 18, 81675 Munich, Germany; 4 Department of Pathology and Immunology, Division of Clinical Pathology, Faculty of Medicine, Centre Médical Universitaire, 1, Rue Michel Servet, 1211 Geneva, Switzerland; 5 Munich Cluster for Systems Neurology, Feodor-Lynen-Str. 17, 81377 Munich, Germany

**Keywords:** NMOSD, EAE, AQP4, MOG, T cells, Autoimmune astrocytopathy

## Abstract

Neuromyelitis optica spectrum disorder (NMOSD) is a paradigmatic autoimmune disease of the central nervous system (CNS), in which the water channel protein Aquaporin-4 (AQP4) is targeted by a self-reactive immune response. While the immunopathology of human NMOSD is largely dependent on antibodies to astrocytic AQP4, the role of AQP4-specific T cells for the localization and quality of NMOSD lesions in the CNS is not known. Only recently, we established that thymic B cells express and present AQP4 in the context of MHC class II molecules to purge the naive T cell receptor repertoire of AQP4-specific clones. Here, we exploited this finding to investigate the lesion localization in the CNS of B cell conditional AQP4-deficient (*Aqp4*^ΔB^) mice, which harbor AQP4-specific precursors in their naive T cell repertoire and can be sensitized to mount a strong AQP4(201–220)-specific CD4^+^ T cell response. Sensitization of *Aqp4*^ΔB^ mice with AQP4(201–220) was sufficient to induce clinical disease. The spatiotemporal lesion distribution and the glial cell response in AQP4(201–220)-induced experimental autoimmune encephalomyelitis (EAE) was compared to classical MOG(35–55)-induced EAE in *Aqp4*^ΔB^ mice. In contrast to MOG-EAE, AQP4(201–220)-induced EAE was characterized by midline lesions in the brain, retinal pathology, and lesions at the grey matter/white matter border zone in the spinal cord. Therefore, we conclude that antigen-specific T cells dictate the localization of NMOSD-lesions in the CNS.

**Supplementary Information:**

The online version contains supplementary material available at 10.1186/s40478-025-01947-8.

## Introduction

Neuromyelitis optica spectrum disorder (NMOSD) is an autoimmune disease of the central nervous system (CNS), in which the water channel protein Aquaporin-4 (AQP4) abundantly expressed on astrocytes is the target antigen [21]. The autoimmune attacks result in severe inflammatory tissue destruction in the spinal cord, optic nerves, and other areas of the CNS [24, 34], which rapidly leads to the accumulation of severe disability if left untreated. Disability is consistently relapse-associated in NMOSD, and, unlike multiple sclerosis (MS), no progressive disease course has been reported, suggesting a different type of tissue response in NMOSD as compared to MS [35].

The immunopathology of NMOSD is largely driven by autoantibodies to AQP4. Anti-AQP4 antibodies bind to astrocytic AQP4, which is structurally organized in so-called orthogonal arrays of particles, resulting in complement-dependent and (perhaps to a lesser degree) antibody-dependent cell-mediated cytotoxic lysis of astrocytes [21]. The localization of NMOSD-lesions in the CNS corresponds to the abundance of AQP4 expression, which is different in distinct CNS niches [19, 24, 31]. While NMOSD lesions in the area postrema are less destructive and have been blamed on the unopposed access of anti-AQP4 antibodies from the serum to the CNS in circumventricular organs where endothelial cells lack tight junctions [27], the development of NMOSD lesions in the spinal cord and optic nerves is not well understood. Early work suggested that the blood–brain barrier in these sites needed to be altered by T cell-mediated inflammation to facilitate the entry of anti-AQP4 antibodies into the CNS parenchyma [3, 4]. In these models, myelin basic protein (MBP)-specific T cells were used to induce subclinical or mild EAE, followed by the transfer of purified patient-derived or monoclonal anti-AQP4 antibodies. Although the pathology in these models resembled human NMOSD lesions [3, 4, 28], the contribution of AQP4-specific T cells to the localization of NMOSD lesions could not possibly be solved in these heterologous systems.

While the contribution of AQP4-specific T cells to lesion development in NMOSD remains to be determined, AQP4-specific T cells are indispensable for the generation of anti-AQP4 antibodies in the systemic compartment. Anti-AQP4 antibodies are hypermutated (affinity matured) and class-switched (IgG1) antibodies that must have gone through a germinal center (GC) reaction where AQP4-specific B cells received help from AQP4-specific T cells [6]. Previously, we and others have reported that T cells are tightly tolerized against AQP4 in the thymus, resulting in the virtual absence of AQP4-specific T cell clones in a healthy naive T cell repertoire [30, 33]. More recently, we established that B cell conditional AQP4-deficient (*Aqp4*^ΔB^) mice harbor AQP4-specific T cells in their T cell repertoire and develop an encephalomyelitis upon immunization with the I-A^b^-restricted AQP4(201–220) epitope [1]. Since *Aqp4*^ΔB^ mice still express AQP4 in the CNS and everywhere else (except in B cells), they represent a prime model to investigate T cell responses against AQP4 and the ensuing immunopathology in different organ systems.

In this study, we characterized the T cell-driven immunopathology against AQP4 and compared it to the T cell-dependent immunopathology against the widely characterized CNS autoantigen myelin oligodendrocyte glycoprotein (MOG) in *Aqp4*^ΔB^ mice. In both cases, we used active immunization against the major I-A^b^-restricted epitopes of MOG and AQP4, i.e. MOG(35–55) and AQP4(201–220), and analyzed the induced experimental autoimmune encephalomyelitis (EAE) clinically, by flow cytometry, and by immunohistochemistry to understand how antigen-specific T cells contribute to lesion localization in NMOSD.

## Material and methods

### Mice

*Aqp4*^flox/flox^ mice [9] were kindly provided by O. P. Ottersen, *Mb1*-Cre mice [11] were kindly provided by M. Schmidt-Supprian, and DEREG mice [15] were kindly provided by T. Sparwasser. All mouse strains were on a C57BL/6J background. Mice were housed in a pathogen‐free facility at the Technical University of Munich. All experimental protocols were approved by the standing committee for experimentation with laboratory animals of the Bavarian state authorities and carried out in accordance with the corresponding guidelines (ROB-55.2-2532.Vet_02-17-234, ROB-55.2-2532.Vet_02-20-01, ROB-55.2-2532.Vet_02-20-23, ROB-55.2-2532.Vet_02-21-154).

### Antigens

Mouse MOG(35–55), MEVGWYRSPFSRVVHLYRNGK, and mouse AQP4(201–220), HLFAINYTGASMNPARSFGP, were synthesized by Auspep (Tullamarine, Australia) or Biotrend (Cologne, Germany), respectively.

### EAE induction

Mice were immunized subcutaneously at the base of the tail with 200 μl of an emulsion containing 200 μg of MOG(35–55) or AQP4(201–220), dissolved in PBS and emulsified with 250 µg *Mycobacterium tuberculosis* H37Ra (BD Difco) in mineral oil (CFA). In addition, mice received 200 ng pertussis toxin (Ptx, Sigma, Cat# P7208) i.v. on days 0 and 2 after immunization. Where indicated, mice received i.v. injections of 20 μg of a monoclonal antibody that recognizes mouse AQP4 (rAb‐53) or control rAb-2B4 against measles virus nucleocapsid protein as described previously [3, 33]. The murine anti‐AQP4 antibody (rAb‐53) was a human-mouse chimeric recombinant antibody (rAb) generated by replacing the human IgG1 Fc region of an AQP4‐specific, NMOSD patient-derived CSF plasma cell clone with a mouse IgG2a Fc region [3]. Both rAb-53 and rAb-2B4 were kind gifts of J. Bennett (Denver). Clinical signs of disease were monitored daily with scores as follows: 0, no disease; 1, loss of tail tone; 2, impaired righting; 3, paralysis of both hind limbs; 4, tetraplegia; 5, moribund state [13]. Clinical data of some *Aqp4*^ΔB^ mice (n = 13 in the MOG(35–55)-immunized group and n = 11 in the AQP4(201–220)-immunized group) were previously published [1].

### Histology

Mice were sacrificed under deep anesthesia by intracardial perfusion with PBS followed by perfusion with 4% w/v paraformaldehyde (PFA) solved in PBS. All organs were removed and fixed in 4% PFA overnight. Vertebral columns, including the spinal cords, were additionally decalcified with Osteosoft® (Sigma-Aldrich) for 72 h before paraffin embedding. To examine the entire CNS, 10–15 sections, each 2 μm thick, with 50 µm intervals apart, were prepared from the spinal cord and brain. In contrast, the optic nerves and eye bulbs were prepared in their entirety with 2 µm thick sections. Immunohistochemistry was performed using a Leica Bond Rxm System with a Polymer Refine detection kit (Leica). A list of all used antibodies is provided in the supplementary material section. DAB was used as chromogen, and counterstaining was performed with hematoxylin. The slides were then scanned on a Leica AT2 system, and the images were analyzed using QuPath v0.3.2 software (https://qupath.github.io, University of Edinburgh, Scotland).

Quantification of histological samples was performed automatically with computer-assisted algorithms provided by QuPath. All annotations were performed in a blinded manner. To detect total cell counts, regions of interest (ROI) were annotated and analyzed automatically by positive nuclear detection. For the semiquantitative analysis, regions containing at least one to five CD45-immunoreactive cells were identified and represented as data points on schemes prepared according to the atlas of Paxinos and Watson [22]. To quantify the loss of AQP4-reactivity, the grey matter areas of axial spinal cord sections were annotated and analyzed by positive pixel detection.

### Isolation of mononuclear cells from CNS

At defined time points, CNS-infiltrating cells were isolated after perfusion through the left cardiac ventricle with PBS. The brain with attached optic nerves and the spinal cord were extracted and digested with collagenase D (2.5 mg/ml) and DNase I (1 mg/ml) at 37 °C for 45 min. After passing the tissue through a 70-μm cell strainer, cells of the spinal cord and brain were separated by discontinuous Percoll gradient (70%/37%) centrifugation. Mononuclear cells were isolated from the interphase for further analyses (flow cytometry).

### Flow cytometry

Single-cell suspensions were incubated with LIVE/DEAD fixable dyes (Aqua [405 nm excitation]) and mouse Fc Block in PBS (phosphate-buffered saline) for 15 min on ice. Cells were washed with fluorescence-activated cell sorting (FACS) buffer (2% FCS in PBS) and incubated with antibodies against surface markers for 30 min on ice. For intracellular cytokine staining, cells were stimulated ex vivo with 50 ng/ml PMA (Sigma-Aldrich, Cat# P1585), 1 μg/ml ionomycin (Sigma-Aldrich, Cat# 10,634), and monensin (1 μl/ml BD GolgiStop, Cat# 554,724) at 37 °C for 2.5 h. Subsequent to LIVE/DEAD and surface staining, cells were fixed and permeabilized (Cytofix/Cytoperm and Perm/Wash Buffer; BD Biosciences, Cat# 554,722 and 554,723) and stained with antibodies against intracellular markers overnight. A list of all antibodies is provided in the supplementary material section.

Flow cytometric analysis was performed on a CytoFLEX flow cytometer (Beckman Coulter) with CytExpert software (v.2.3.1.22), and flow cytometric data were analyzed using FlowJo software (v10.5.1, BD Biosciences).

### Statistical analysis

Statistical evaluations of cell frequency measurements and cell numbers were performed with one-way-ANOVA and post hoc tests when more than two populations were compared. Two-way ANOVA followed by post hoc multiple comparison tests was used, as indicated in the figure legends. EAE incidence was calculated using Kaplan–Meier analysis, and the P-values were analyzed using a log-rank test (Mantel-Cox). A P-value of P < 0.05 was considered significant. Calculations and the generation of graphs were performed using Graph Pad Prism v10.9.0 (GraphPad software). Figures were prepared with Adobe Illustrator 2022 (v.26.0.1).

### Licenses

In Supp. Figure 1a pictures from Servier Medical Art were used that are licensed under a Creative Commons Attribution 3.0 Unported License (https://creativecommons.org/licenses/by/3.0/).

## Results

### Inflammatory infiltrates affect AQP4-rich CNS niches in AQP4(201–220)-induced EAE

**Fig. 1:**
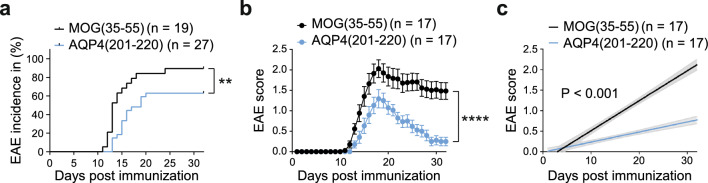
Incidence and disease severity in MOG(35–55)-induced vs. AQP4(201–220)-induced EAE in *Aqp4*^ΔB^ mice. **a** EAE incidence and **b** EAE severity (mean ± s.e.m.) in MOG- versus AQP4-peptide-induced EAE. **c** Simple linear regression analysis of disease scores of clinically sick MOG(35–55)- and AQP4(201–220)-immunized *Aqp4*^ΔB^ mice. Statistical analysis was performed using Mantel-Cox log-rank tests and two-way ANOVA with Sidak’s post-test to compare incidences (**a**) and disease course (**b**), respectively

The absence of AQP4 expression in B cells allows AQP4-specific T cells to seed secondary lymphoid tissues, thereby creating a sufficiently large precursor frequency to elicit a productive T cell response upon immunization with the AQP4(201–220) peptide [1]. As a consequence, *Aqp4*^ΔB^ mice are susceptible to AQP4(201–220)-induced EAE. Since *Aqp4*^ΔB^ mice are also susceptible to conventional MOG(35–55)-induced EAE, we could directly compare the clinical and histopathological phenotype of these two diseases. The two disease models exhibit a comparable phenotype, with motor deficits suggestive of a spinal cord syndrome affecting the tail and limbs in a symmetrical manner (Fig. 1a, b). Following immunization, mice started exhibiting motor deficits on day 11 that peaked around day 18. A subsequent recovery period spanned until day 30 (Fig. 1b). Incidence and disease severity according to the established EAE scoring scale were significantly higher in MOG(35–55)-induced EAE than in AQP4(201–220)-induced EAE (Fig. 1a–c).

To further investigate whether the inflammatory infiltrates in the CNS were different in MOG vs. AQP4-induced EAE, we performed flow cytometric analyses of the CNS infiltrating immune cells. Here, we did not observe significant differences in the composition or functional phenotype of the mononuclear cell infiltrates either in the brain or in the spinal cord of MOG-induced vs. AQP4-induced EAE (Supplementary Fig. 1).

**Fig. 2:**
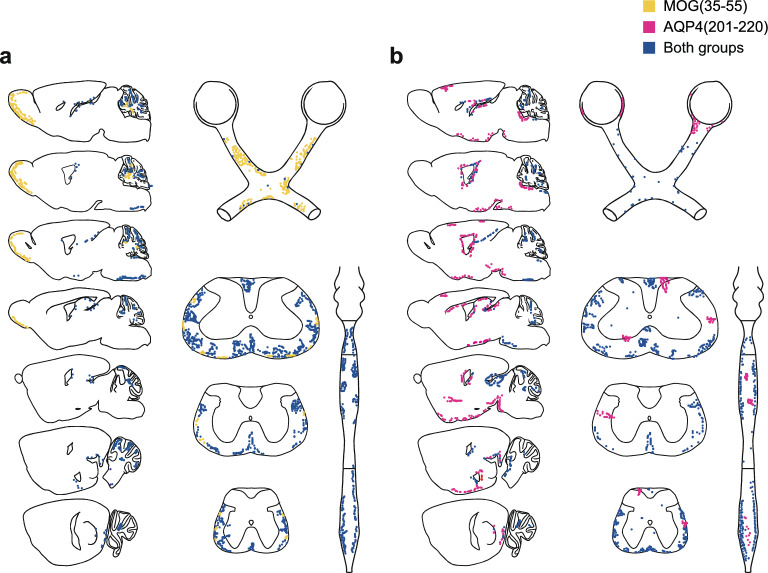
Lesion topology in the CNS in AQP4(201–220)-induced EAE at the peak of disease. Semiquantitative analysis of the lesion distribution in the CNS at the peak (d2 after disease onset) of **a** MOG(35–55)- and **b** AQP4(201–220)-induced EAE. Schemes were prepared according to Paxinos and Watson [22]. Each data point represents a formation containing at least one to five CD45-immunoreactive cells as identified by QuPath automated cell detection. The coloring indicates whether the detection was present in a single group or in both, as specified in the legend

Since the EAE score is biased toward detecting spinal cord disease, the clinical phenotype may miss alternative lesion locations in AQP4-induced EAE. Therefore, we performed an extensive immunohistochemical workup of all CNS compartments, including the retina and the optic nerve, in AQP4-induced versus MOG-induced EAE. Individual EAE mice with a score greater than 2.0 were analyzed when they reached the peak of disease according to the conventional EAE score and during recovery (on d32 after immunization). Lesions were defined as aggregates of CD45-reactive cells in the forebrain, cerebellum, spinal cord, olfactory bulb, optic nerve, and retina. At the peak of clinical disease (d15–d18), MOG(35–55)-induced EAE-mice demonstrated extensive sharply delineated mononuclear cell infiltrates across the cerebellum, spinal cord, olfactory bulb, and optic nerves (Fig. 2a). In AQP4(201–220)-induced EAE, we observed longitudinal, scattered lesions in the white matter of the lumbar and cervicothoracic spinal cord, often localized in the grey matter/white matter border zone. In addition, inflammatory lesions were frequently found in the diencephalon and in the retina in AQP4-induced EAE. In contrast to the lesion distribution in AQP4-induced EAE, lesions were very rare in the diencephalon and essentially absent in the retina in MOG-induced EAE (Fig. 2a, b). In summary, the localization of inflammatory infiltrates was largely consistent with the expression of MOG and AQP4 expression in different CNS compartments [31]. In contrast to MOG, AQP4 is abundantly expressed in peripheral tissues such as the kidney. Here, we noticed infiltrates of CD45^+^ cells in the kidney parenchyma of one out of five AQP4(201–220)-immunized mice but in none of the MOG(35–55)-immunized animals (Supplementary Fig. 2).

As reflected by the clinical score, the recovery from spinal cord disease was more pronounced in AQP4(201–220)-induced EAE than in MOG(35–55)-induced EAE in *Aqp4*^ΔB^ mice (Fig. 1). Similar to the spinal cord, the persistence of CD45^+^ infiltrates in brain sites was shorter after AQP4(201–220)-immunization than after MOG(35–55)-immunization, suggesting that inflammation per se is well cleared after an AQP4 targeted T cell response, even in the retina and the optic nerve (Supplementary Fig. 3).

### Immunopathology and tissue response in MOG(35–55)- versus AQP4(201–220)-induced EAE in *Aqp4*^ΔB^ mice

Autoimmunity directed against AQP4 affected overlapping and distinct CNS niches as compared with autoimmunity directed against MOG. However, due to the T-cell-driven inflammatory response in our peptide-induced disease model, the composition and quality of the inflammatory infiltrates were very similar in MOG-induced and AQP4-induced EAE. To assess the configuration of individual inflammatory lesions and the glial response they elicited, we analyzed the tissue reactivity to Iba-1, LFP-PAS, AQP4, and GFAP, together with staining for CD45 in serial sections.

**Fig. 3:**
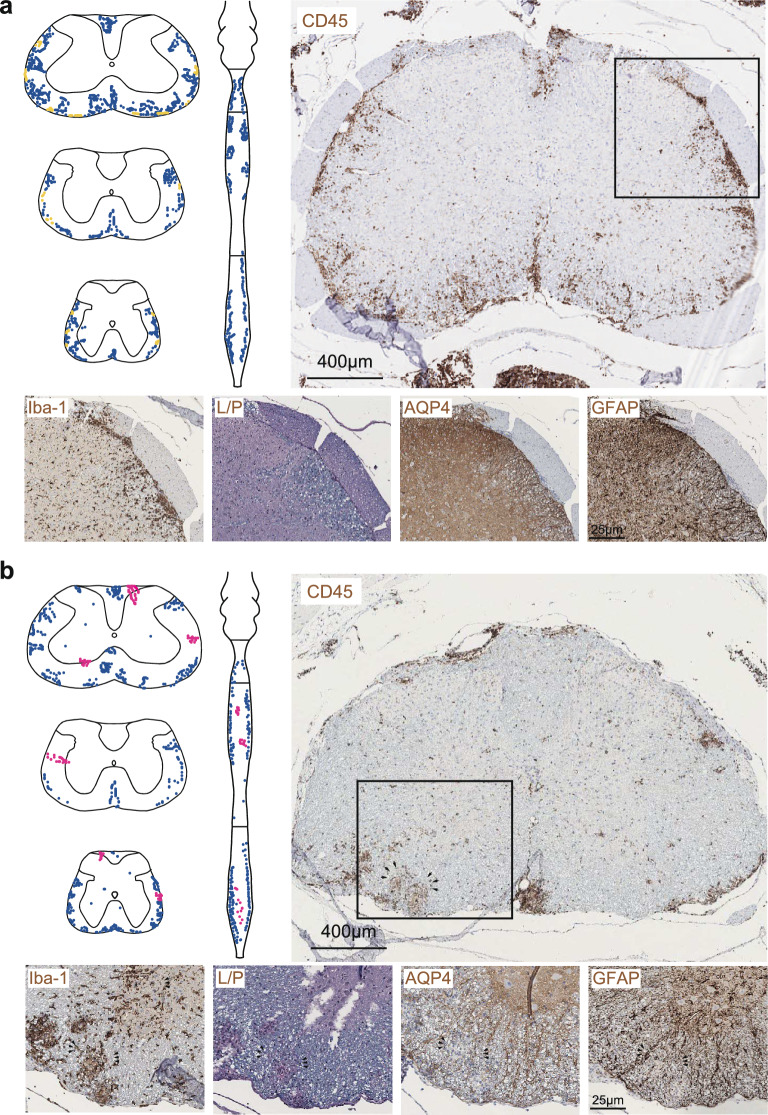
Spinal cord pathology in MOG(35–55)-induced versus AQP4(201–220)-induced EAE. Representative immunostaining for CD45, Iba-1, LFB-PAS (L/P), AQP4, and GFAP in spinal cord sections obtained from **a** MOG(35–55)- and **b** AQP4(201–220)-immunized *Aqp4*^ΔB^ mice at the peak of EAE (n = 3 independent experiments). Scale bars **a**, **b** 400 µm (top) and 25 µm in enlarged image sections

In the spinal cord, CD45-immunoreactive lesions in MOG-induced EAE were mostly located in the dorsal and anterolateral white matter tracts. These lesions formed dense and confluent formations that had contact with the meningeal compartment (Fig. 3a). In AQP4(201–220)-induced EAE, a patchy pattern of CD45-immunoreactive lesions was observed, occurring in two distinct localizations: Scattered formations that had contact with the meninges and delineated lesions at the border zone between grey and white matter (Fig. 3b). Demyelination and reactivity of microglia (Iba-1) and astrocytes (GFAP) were more pronounced in the spinal cords of mice with MOG(35–55)-induced EAE in contrast to AQP4(201–220)-induced EAE, where AQP4 loss was a characteristic feature (Fig. 3a, b).

**Fig. 4:**
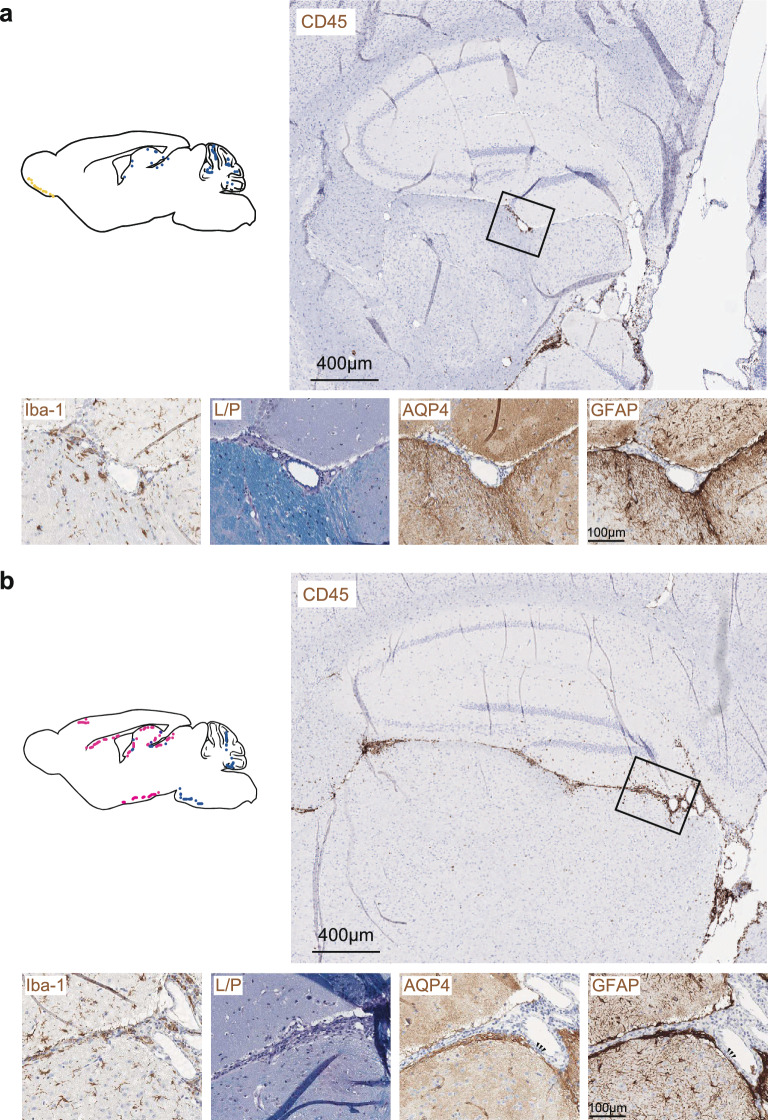
Topology and pathology of brain lesions in MOG(35–55)-induced vs. AQP4(201–220)-induced EAE. Representative immunostaining for CD45, Iba-1, LFB-PAS (L/P), AQP4, and GFAP in the brains (sagittal section) of *Aqp4*^ΔB^ mice with **a** MOG(35–55)-induced and **b** AQP4(201–220)-induced EAE at the peak of disease (n = 3 independent experiments). Scale bars 400 µm (top) and 100 µm in enlarged image sections

In the brain, distinct from MOG-induced EAE (Fig. 4a), a key feature of AQP4(201–220)-induced EAE was the localization of inflammatory lesions in the midline of the diencephalon in proximity to the third ventricle and also around the fourth ventricle (Fig. 4b)—lesion sites that are consistent with what was described in human NMOSD [23, 24]. Lesions were characterized by loss of AQP4 reactivity (Fig. 4b). In contrast, these CNS niches were hardly affected in MOG(35–55)-induced EAE, and sites of inflammatory lesions in MOG-induced EAE did not lose AQP4-reactivity although astrocyte reactivity and microgliosis was uniformly more pronounced in MOG-induced than in AQP4-induced EAE (Fig. 4a, b).

**Fig. 5:**
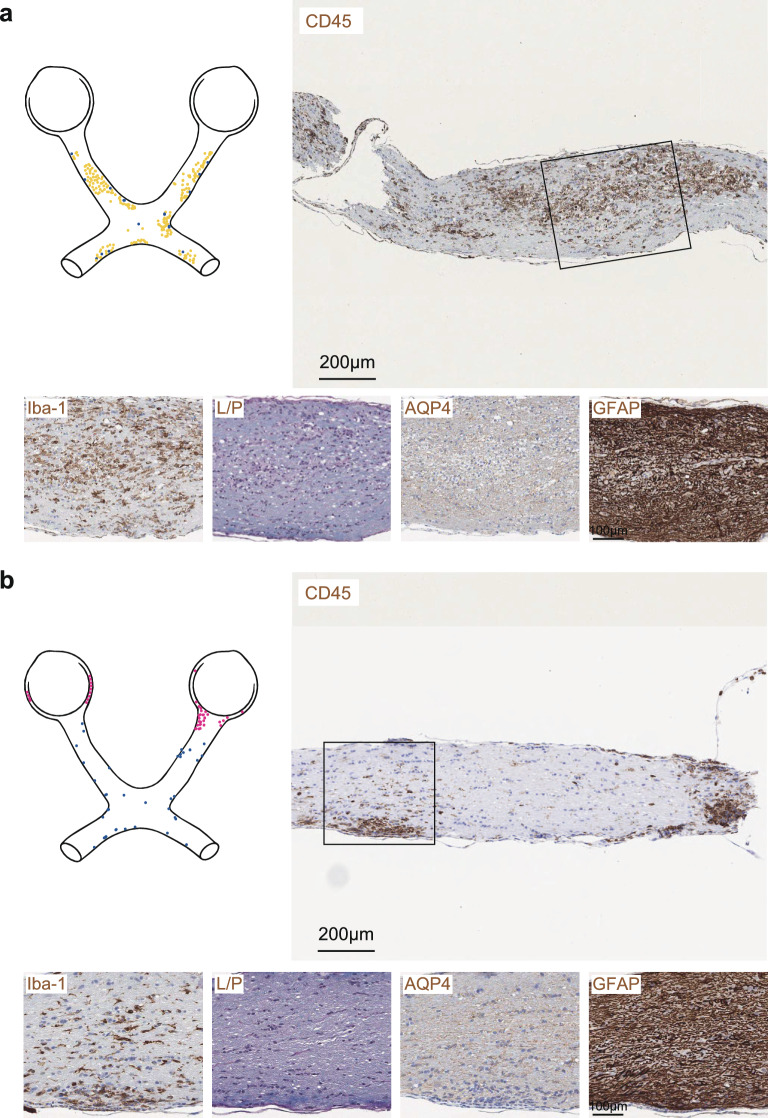
Pathology of optic nerve lesions in MOG(35–55)-induced vs. AQP4(201–220)-induced EAE. Representative immunostaining for CD45, Iba-1, LFB-PAS (L/P), AQP4, and GFAP in the optic nerves of *Aqp4*^ΔB^ mice with **a** MOG(35–55)-induced and **b** AQP4(201–220)-induced EAE at the peak of disease (n = 3 independent experiments). Scale bars 200 µm (top) and 100 µm in enlarged image sections

The lesion pattern in the optic nerves was also distinct in MOG-induced vs. AQP4-induced EAE. While optic nerve lesions in MOG-induced EAE often included perineural areas and were widespread along nerve fibers in the optic nerve parenchyma, optic nerve lesions in AQP4-induced EAE were patchy (Fig. 5a, b). Optic nerves in MOG(35–55)-induced EAE exhibited a robust glial response, with pronounced Iba-1 and GFAP reactivity, accompanied by extensive demyelination (Fig. 5a). While demyelination and reactive astrocytes were also present in the optic nerves after AQP4(201–220)-immunization, the overall inflammatory response was less pronounced than in MOG(35–55)-induced EAE (Fig. 5b).

**Fig. 6:**
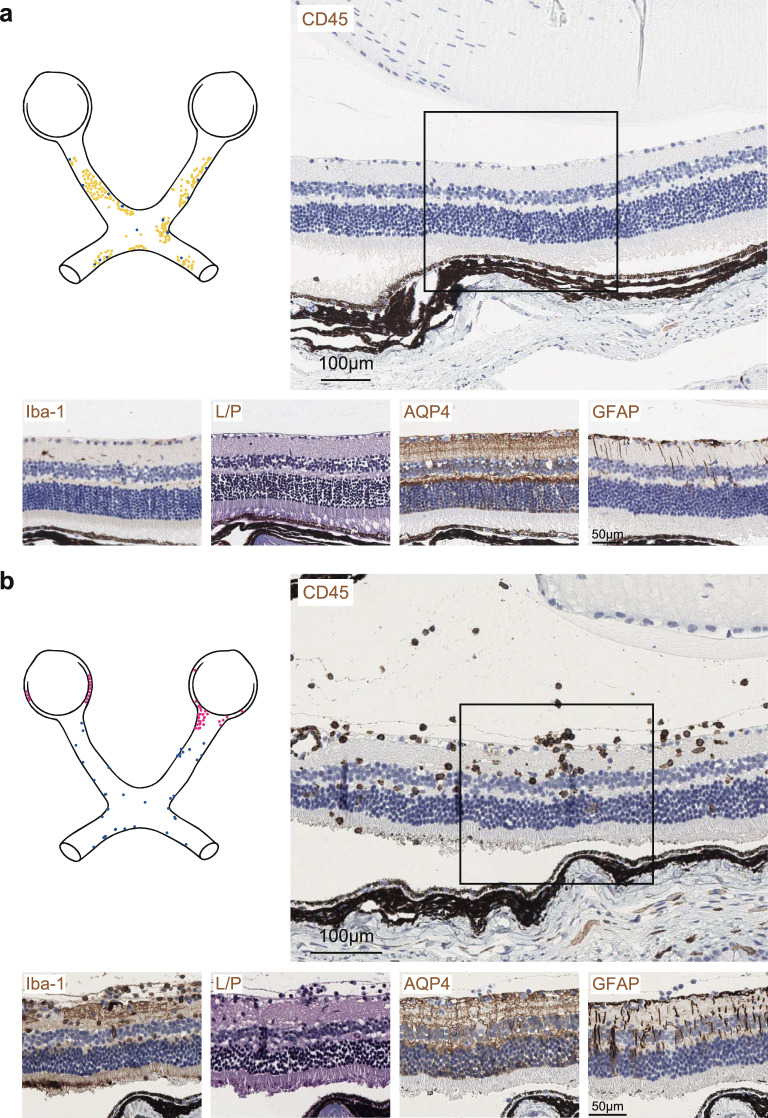
Retinal pathology in MOG(35–55)-induced vs. AQP4(201–220)-induced EAE. Representative immunostaining for CD45, Iba-1, LFB-PAS (L/P), AQP4, and GFAP in the retinas of *Aqp4*^ΔB^ mice with **a** MOG(35–55)-induced and **b** AQP4(201–220)-induced EAE at the peak of disease (n = 3 independent experiments). Scale bars 100 µm (top) and 50 µm in marked image sections

The most notable difference between MOG-peptide and AQP4-peptide-induced EAE in *Aqp4*^ΔB^ mice was observed in the retina. As previously reported by us and others, retinal changes are a hallmark of MOG(35–55)-induced EAE [2, 18]. These changes include reactive astrocytosis and retinal ganglion cell loss. However, direct inflammatory infiltrates of hematopoietic cells were never observed in the retina of MOG-EAE mice (Fig. 6a). In contrast, we observed CD45^+^ immune cell infiltration in the inner retinal layers accompanied by significant AQP4 loss and microglial activation in AQP4-EAE (Fig. 6b). These lesions resulted in ganglion cell loss.

In summary, spinal cord grey matter/white matter border lesions, midline lesions in the diencephalon, and loss of AQP4-immunoreactivity in the proximity of inflammatory lesions were unique to AQP4-induced EAE. Reactive astrocytosis, as assessed by GFAP-reactivity, and microglia activation (Iba-1 signal) were generally more widespread in MOG-induced EAE than in AQP4-induced EAE. In the recovery stage (around d32), the glial response was still observed in the spinal cord, cerebellum, and optic nerves of MOG-EAE. In contrast, the tissue response in AQP4-EAE was largely cleared by d32, and even AQP4-reactivity was restored in areas most affected in AQP4-EAE at the peak of the disease (Supplementary Figs. 4–7).

**Fig. 7:**
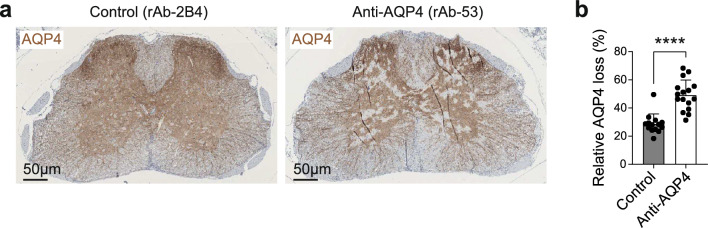
The extent of the CNS damage in NMOSD depends on the presence of anti-AQP4 antibodies. **a** Representative immunostaining for AQP4 and **b** quantification of AQP4 loss in the grey matter of transverse spinal cord sections of *Aqp4*^ΔB^ mice immunized with AQP4(201–220) and treated with either control antibody (rAb-2B4) or anti-AQP4-antibody (rAb-53). Mice received i.v. injections of 20 μg of the respective monoclonal antibodies at day 0 and 2 after disease onset. One day after the last injection, mice were sacrificed and perfused for histological work-up. AQP4 loss was defined as 1—positively detected (AQP4-reactive) pixels per region of interest (ROI) area (grey matter). Scale bars 50 µm, data shown as mean ± s.d. Multiple spinal cord sections of two control-treated and two rAb-53-treated mice were analyzed. Statistical analysis was performed using two-tailed unpaired t-tests. *P < 0.05

Although AQP4(201–220) peptide-immunized mice do not raise an anti-AQP4 antibody response [33], we observed some lesion-associated loss of AQP4-reactivity in AQP4(201–220)-immunized *Aqp4*^ΔB^ mice, suggesting that an antigen-specific T cell response alone was sufficient to induce loss of AQP4-reactivity in AQP4(201–220)-induced EAE. To gauge the additional contribution of anti-AQP4 antibodies to the pathology of AQP4^+^ astrocytes in this model, we immunized *Aqp4*^ΔB^ mice with AQP4(201–220) and, upon first clinical signs of disease, treated them with two injections of a control antibody (rAb-2B4) or a pathogenic anti-AQP4 antibody that recognizes an extracellular epitope of AQP4 (rAb-53, [33]). The loss of AQP4-immunoreactivity in white and grey matter (as detected by an antibody directed against the C-terminal intracellular domain of AQP4) was significantly more extensive in anti-AQP4-treated than in control-treated mice (Fig. 7a, b). In particular, in the cervical and lumbar spinal cord grey matter where AQP4-peptide immunized *Aqp4*^ΔB^ mice showed only small lesions (Figs. 2b**, **b), these lesions were markedly enlarged upon additional administration of rAb-53 (Fig. 7a, b). Therefore, while an AQP4-specific T-cell response alone can induce pathology to AQP4^+^ astrocytes, the structural damage is largely amplified in the additional presence of AQP4-specific antibodies.

## Discussion

Activated AQP4(201–220)-specific T cells produce a retino-optico-diencephalo-spinal autoimmune inflammatory syndrome, which differs from MOG(35–55)-induced EAE as to lesion localization, intensity of inflammation, and glial cell response. Direct inflammatory infiltrates in the retina and in midline-associated areas of the diencephalon were only found in AQP4(201–220)-induced EAE while absent in MOG(35–55)-induced EAE. In the spinal cord, AQP4(201–220)-induced lesions were located at the white matter/grey matter border zone, whereas MOG(35–55)-induced inflammation emerged from the meningeal compartment and extended into white matter tracts. Overall, reactive astrocytosis and microglial activation appeared to be less pronounced in AQP4(201–220)-induced than in MOG(35–55)-induced EAE. Consistently, in remission, immunopathology was essentially cleared in AQP(201–220)-induced EAE. Therefore, AQP4(201–220)-specific T cells determine a different lesion topology as compared with MOG(35–55)-induced EAE but have per se (in the absence of an anti-AQP4-antibody response) a lower potential than MOG(35–55)-specific T cells to promote sustained tissue inflammation.

While EAE was induced by adoptive transfer of in vitro activated AQP4-specific T cells [12, 36], it has been impossible to investigate the encephalitogenic potential of AQP4-specific T cells in an active immunization model since the T cell repertoire is essentially purged of AQP4-specific precursors in naive wild-type animals. Thymic tolerance largely determines the loss of AQP4-reactive clones in the T cell compartment [1, 33]. However, in a scenario where thymic tolerance to AQP4 is broken (by genetic ablation of *Aqp4* in B cells [1]), additional layers of T cell tolerance to AQP4 might be operational [29]. While *Aqp4*^ΔB^ mice exhibit an inflammatory AQP4(201–220) T cell response, they also harbor AQP4-specific Foxp3^+^ Treg cells. Therefore, peripheral AQP4-specific T-cell tolerance awaits further analysis, in particular, because AQP4 is an autoantigen that is widely expressed in many tissues. Differences in the regulation of the I-A^b^ restricted T cell response to AQP4 vs. MOG may also partially explain the differences in disease incidence and inflammatory burden in AQP4(201–220)-immunized vs. MOG(35–55)-immunized *Aqp4*^ΔB^ mice [29].

The immunopathology of MOG(35–55)-induced EAE has meticulously been characterized in its different stages [5]. This analysis supported the idea that the inflammatory process in MOG(35–55)-induced EAE starts in the meningeal compartment and extends into the CNS parenchyma across the glia limitans but at the same time induces distal activation of the deep parenchymal vascular compartment as well as microglial activation. Accordingly, CNS niches most exposed to the CSF space (like the spinal cord and the middle cerebellar peduncle) were the first to be affected by inflammatory alterations and showed distal axonal damage in MOG(35–55)-induced EAE [5]. The lesion pattern of MOG(35–55)-induced EAE in *Aqp4*^ΔB^ mice is consistent with this report, including the observation that the olfactory bulb and tract were strongly affected by inflammatory infiltrates.

Reports of actively induced AQP4-EAE have essentially been lacking due to the resistance of C57BL/6 mice to mount AQP4-specific T-cell responses [33]. Yet, it has been shown that an encephalomyelitic syndrome can be evoked in mice by adoptive transfer of AQP4-specific T cells derived from *Aqp4*^–/–^ mice [12, 30, 33]. AQP4(201–220) is the major I-A^b^-restricted immunogenic epitope of AQP4 [30, 33]. While immunization of *Aqp4*^–/–^ mice with AQP4(135–153) also elicits an AQP4-specific T cell response [12], AQP4(135–153), which is a peptide comprised in the C-loop of AQP4, is unlikely to be a naturally processed epitope of AQP4 since immunization of *Aqp4*^–/–^ mice with full-length AQP4 protein fails to elicit an AQP4(135–153)-specific response while an AQP4(201–220)-specific T cell response can readily be recalled [33]. The immunopathology of EAE, in which AQP4 is targeted, has only been described in a setting where AQP4(201–220)-specific or AQP4(135–153)-specific *Aqp4*^–/–^ T cells were differentiated ex vivo into Th17 cells and then adoptively transferred into naive recipient mice [12, 30]. The spinal cord and optic nerve, but not other areas of the CNS, were analyzed and exhibited inflammatory infiltrates with a focus on the meningeal compartment [30]. Little or no loss of AQP4 reactivity in astrocytes is observed upon transfer of AQP4(135–153)-specific T cells [12]. While the quality of the inflammatory infiltrates in these adoptive transfer models is consistent with our results, the comparability to our model in terms of the dynamics and topology of lesions is limited since we elicited EAE with a natural epitope in a polyclonal T cell repertoire by active immunization. In fact, wild-type mice are resistant to AQP4(201–220)-induced EAE due to efficient thymic deletion of AQP4-specific precursor T cells. Conversely, while global *Aqp4*^–/–^ mice develop robust AQP4-specific T-cell responses, they cannot serve as a disease model since they lack the target antigen in the CNS [1, 33].

An extensive histological workup of EAE induced by AQP4-specific T cells has also been performed in Lewis rats [36]. Here again, AQP4-specific T cells were adoptively transferred into naive Lewis rat recipients. AQP4(268–285) was identified to be an immunogenic and naturally processed epitope of AQP4 in the context of RT1.B^L^ (the Lewis rat MHC class II complex). Notably, the localization of lesions induced by adoptive transfer of AQP4(268–285)-specific T cells in Lewis rats, in particular in the retina, around the third ventricle and at the white matter/grey matter border zone in the spinal cord, was similar to what we observed in our AQP4(201–220)-induced EAE [36, 37].

Our model cannot be a faithful model of human NMOSD since it lacks an intrinsic anti-AQP4 antibody response, which is the major limitation of the present model. However, we provide evidence that an AQP4-specific T cell response evoked from a natural polyclonal T cell repertoire is able to induce encephalomyelitis at sites that recapitulate the lesion localization in human NMOSD. Therefore, T cells alone are sufficient to reproduce the topology of NMOSD lesions in the CNS, suggesting that AQP4-specific T cells are involved in dictating the hot spots of immunopathology in NMOSD. The i.v. administration of a murinized monoclonal anti-AQP4-antibody [33] to AQP4(201–220)-immunized *Aqp4*^ΔB^ mice markedly enlarged the loss of AQP4-reactivity in all localizations with T cell-dependent inflammatory infiltrates, in particular in the cervical and lumbar spinal cord grey matter. Since inflammatory lesions (albeit much smaller) were also found at these sites in the absence of anti-AQP4 antibodies, antigen-specific T cells are an important determinant of the lesion topology in this NMOSD model. This observation is consistent with previous studies that highlight the significance of antigen-specific T cells for instructing the sites of anti-AQP4 antibody-induced lesions depending on the expression pattern of the respective T cell target antigen [14, 25, 26]. Nevertheless, the extent of individual lesions in NMOSD and—in the case of very high-affinity anti-AQP4 antibodies—perhaps also the localization of NMOSD lesions is driven by anti-AQP4 antibodies alone with no or only minor contribution of antigen-specific T cells [10, 20]. The anti-AQP4 antibody used in the present study (rAb-53) was engineered by replacing the human IgG1-Fc with a mouse IgG2a-Fc in a human NMOSD CSF plasma cell-derived clone that recognizes both human and mouse AQP4 [3, 33]. NMOSD patient-derived anti-AQP4 antibodies preferentially recognize the M23 isoform of AQP4 that forms orthogonal arrays of particles in astrocytes [7, 20]. The affinity to these conformational AQP4 epitopes is a determinant of their effector function in terms of complement binding and, thus, extension of lesions [32].

In the absence of an anti-AQP4 antibody response, T cell-mediated pathology was more severe in MOG(35–55)-induced EAE than in AQP4(201–220)-induced EAE, although, in both scenarios, the CFA-dependent adjuvant effect resulted in a strong systemic T cell activation. While differences in the immune regulation of the T cell response against these two autoantigens might exist (see above), differences in the tissue response in the CNS might also contribute to the differences in immunopathology. MOG(35–55)-induced EAE is characterized by oligodendrocyte apoptosis and extensive inflammatory axonal damage. In contrast, AQP4(201–220)-induced EAE targets astrocytes that might have greater regenerative potential unless secondary damage largely mediated by anti-AQP4 antibodies with complement-mediated effector functions (that were lacking in our model) also affects oligodendrocytes and axons. In fact, in human NMOSD, the quality of the CNS damage depends on anti-AQP4 antibodies [28, 36]. Extremely high-affinity anti-AQP4 antibodies might even be able to establish NMOSD lesions in the absence of activated T cells in the CNS compartment [10].

In summary, AQP4(201–220)-induced EAE in *Aqp4*^ΔB^ is a useful tool for investigating the effector functions of AQP4-specific T cells in CNS autoimmunity. This model provides an experimental platform to investigate tissue responses in the CNS when an astrocytic antigen is directly targeted as compared to antigens (like MOG) that are expressed in oligodendrocytes. For instance, it is unclear whether astrocytes are able to present AQP4 epitopes in the context of MHC class II molecules in an inflamed environment. Conversely, the astrocyte response might be different when they are a direct target of an autoimmune reaction as compared to a scenario where they are bystanders to an oligodendrocyte-directed attack. This aligns with the observed difference in microglial activation between MOG- and AQP4-induced EAE. There is a growing body of evidence supporting the critical involvement of astrocytes during inflammatory insults to the CNS [8, 17]. Astrocytes might even exhibit an immunologic memory mediated by epigenetic modification, licensing them as competent mediators of CNS inflammation [16]. Considering the differential glial composition of distinct CNS compartments with specialized glial cell subsets such as retinal Müller cells, modeling of astrocyte-specific autoimmunity will provide novel avenues to better understand compartmentalized inflammation to develop tailored therapeutic interventions in a situation where astrocytic and not oligodendroglial antigens are directly targeted by a specific autoimmune response.

## Supplementary Information

Additional file 1.

Additional file 2.

## Data Availability

The datasets used and/or analyzed during the current study are available from the corresponding author on reasonable request.

## Abbreviations

AQP4: Aquaporin-4
*Aqp4*^Δ^^B^: B cell conditional AQP4-deficient
CEP: Comparative Experimental Pathology
CNS: Central nervous system
DFG: Deutsche Forschungsgemeinschaft
EAE: Experimental autoimmune encephalomyelitis
FACS: Fluorescence-activated cell sorting
GC: Germinal center
L/P: LFB-PAS
MBP: Myelin basic protein
MOG: Myelin oligodendrocyte glycoprotein
MS: Multiple sclerosis
NMOSD: Neuromyelitis optica spectrum disorder
PFA: Paraformaldehyde
ROI: Region of interest
TCR: T cell receptor
Treg cell: Regulatory T cell
