# Supplementary figures and images for: AQP4-specific T cells determine lesion localization in the CNS in a model of NMOSD
Source: Acta Neuropathol Commun. 2025 Feb 11;13:27. doi: 10.1186/s40478-025-01947-8 (PMC11817536; doi:10.1186/s40478-025-01947-8)

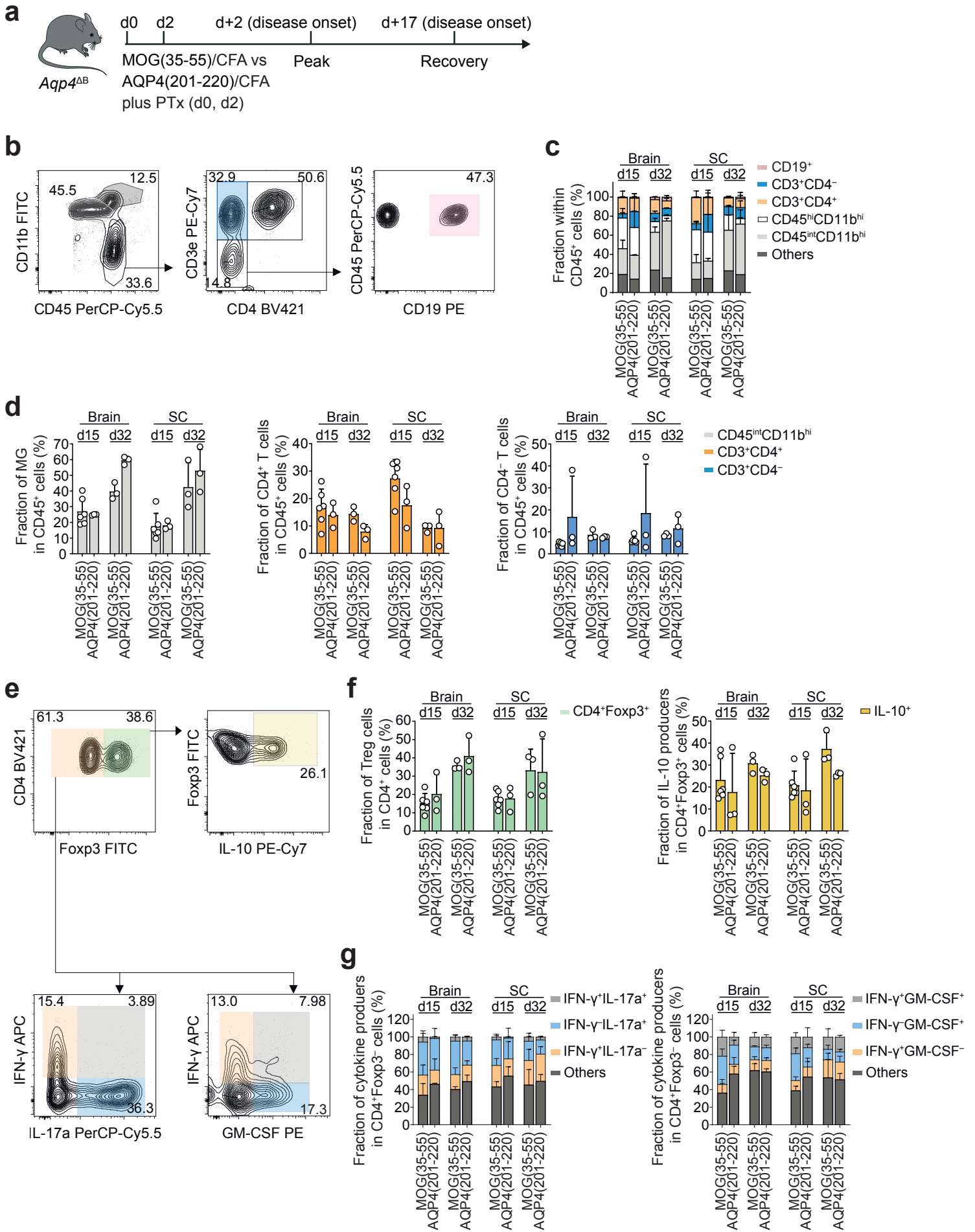

**a**

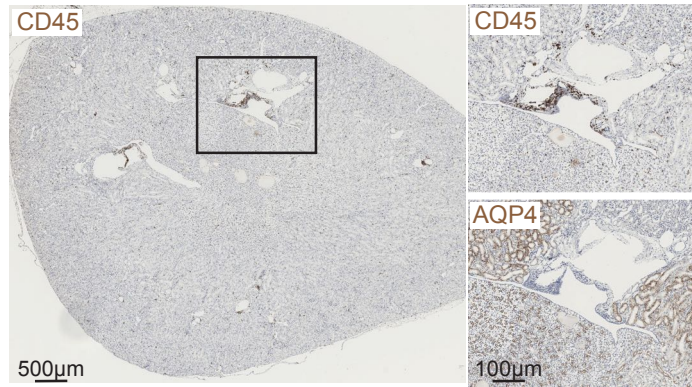

**b**

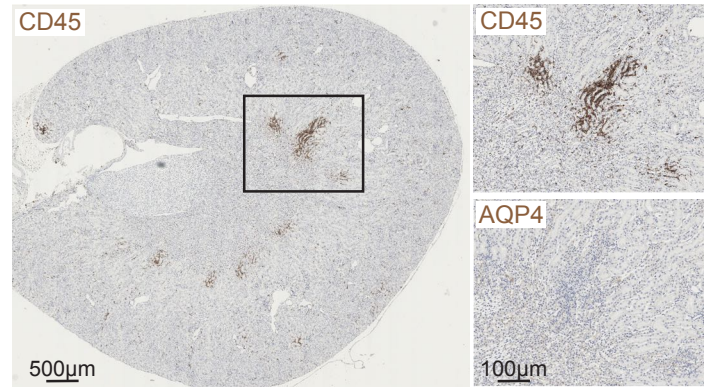

Supplementary Figure 3

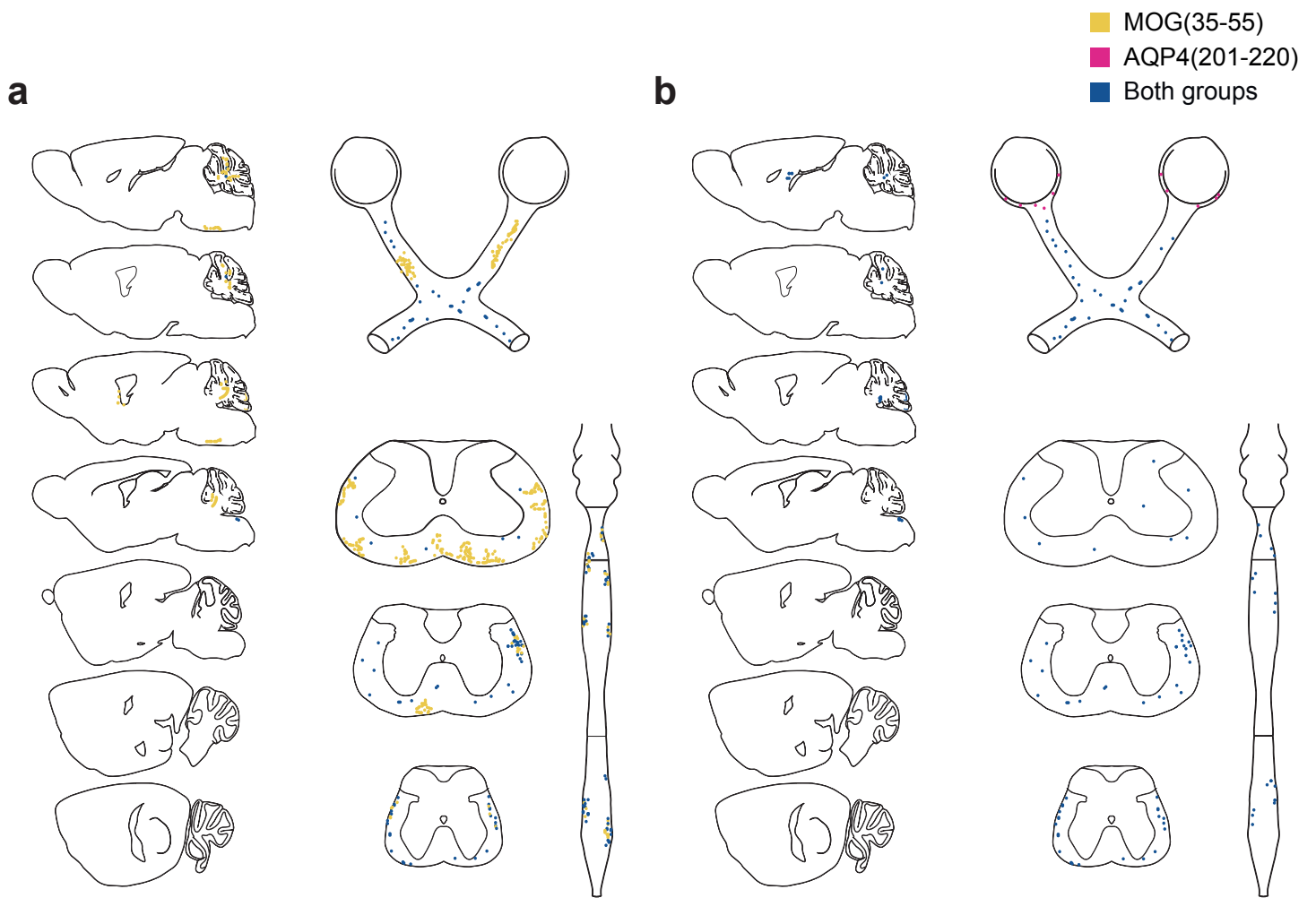

Supplementary Figure 4

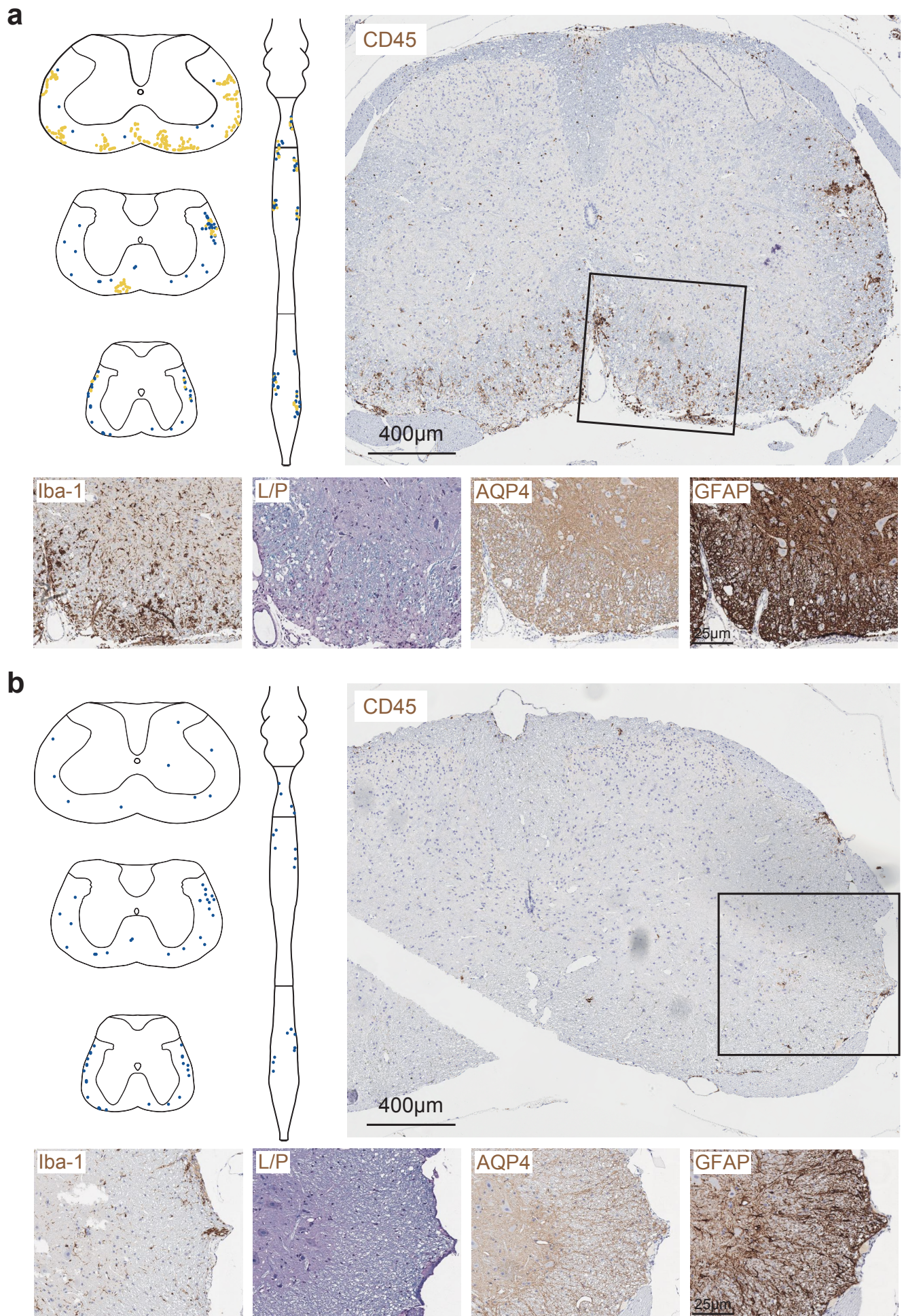

**a**

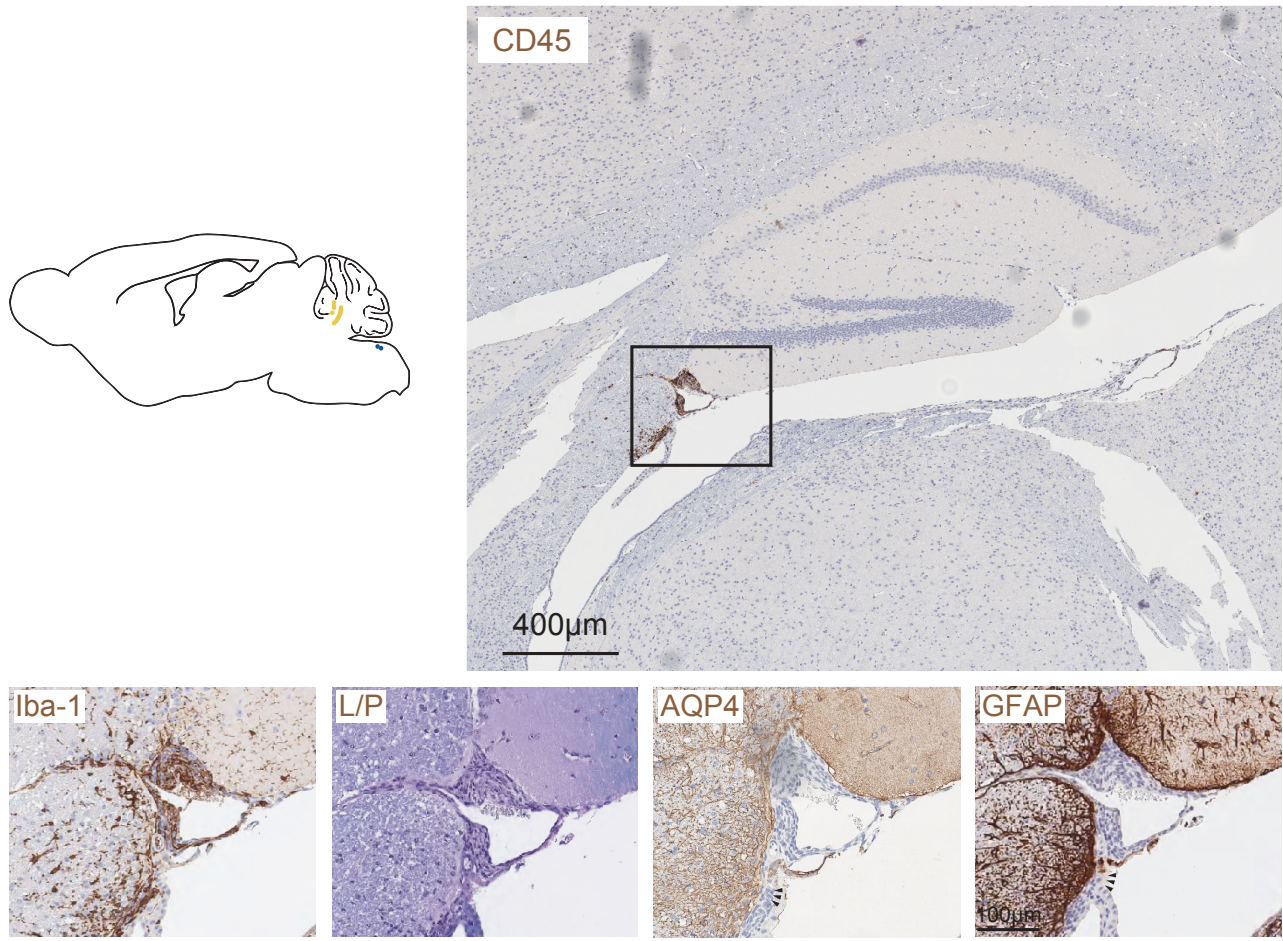

**b**

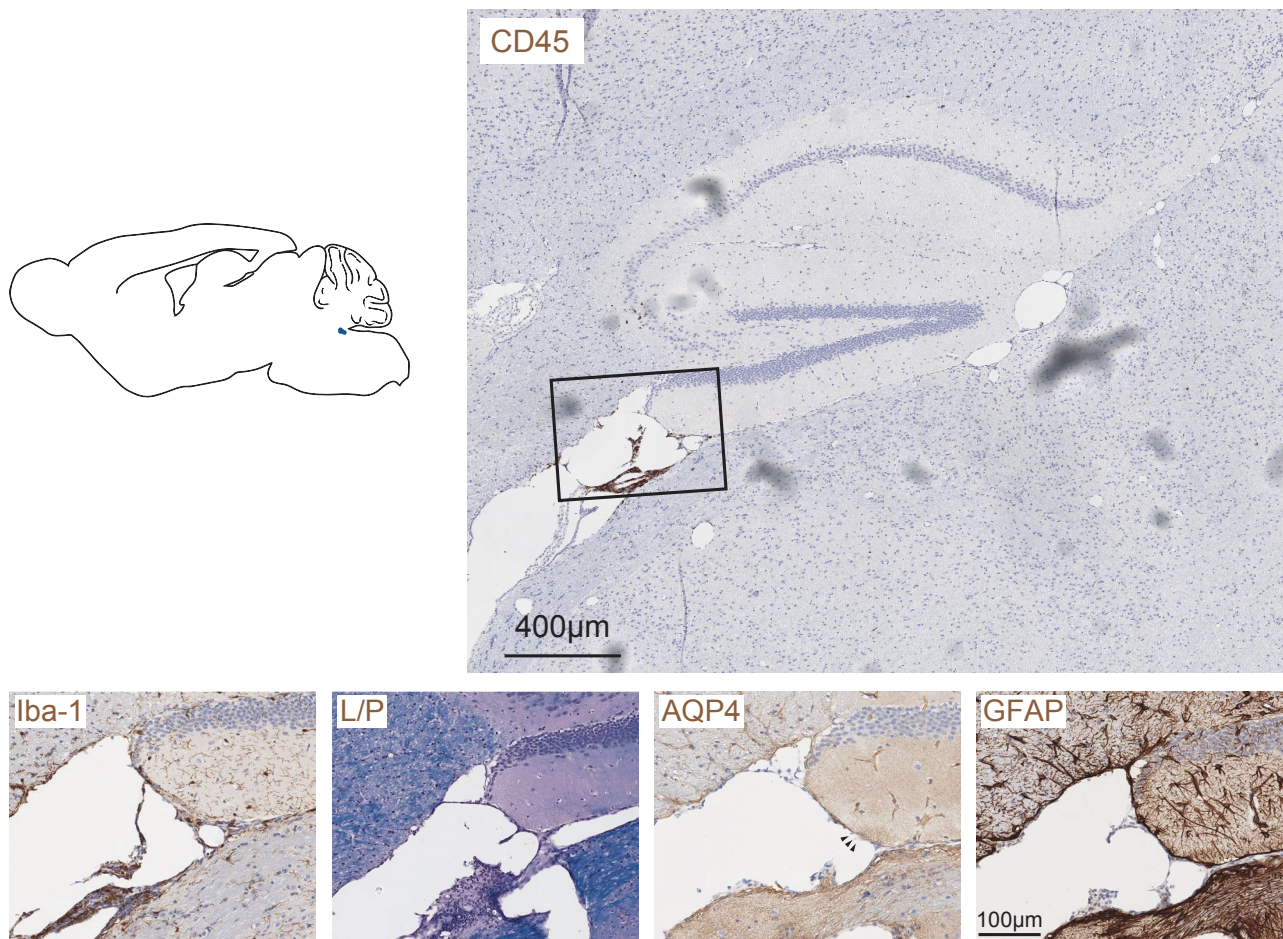

**a**

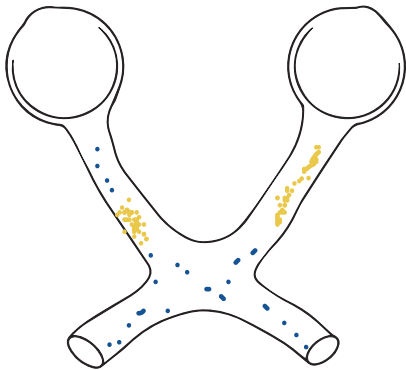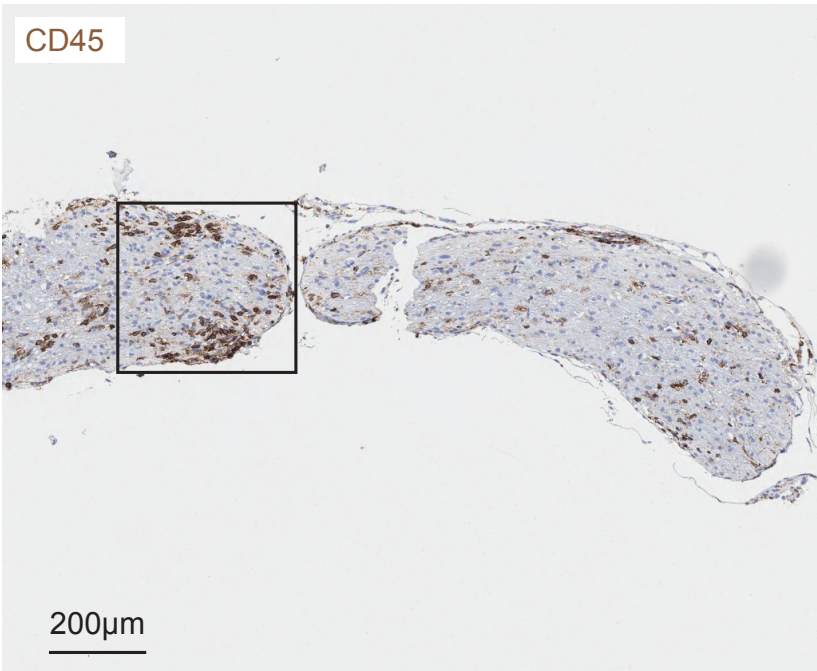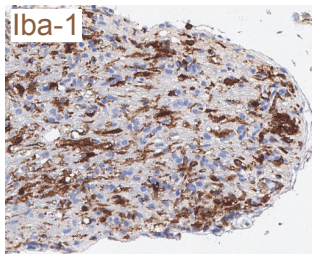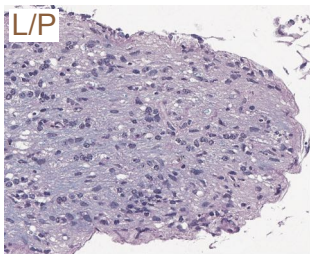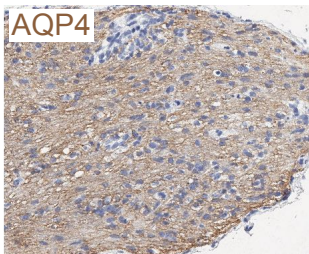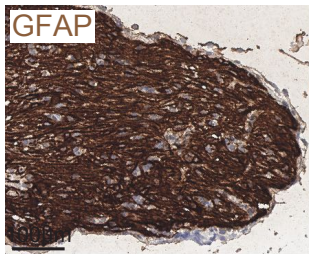

**b**

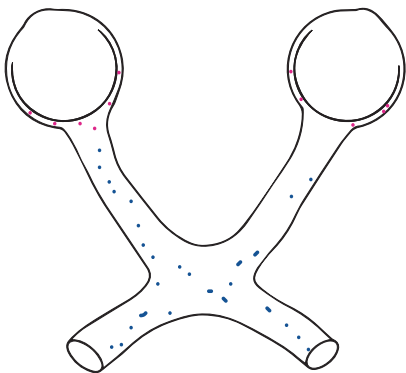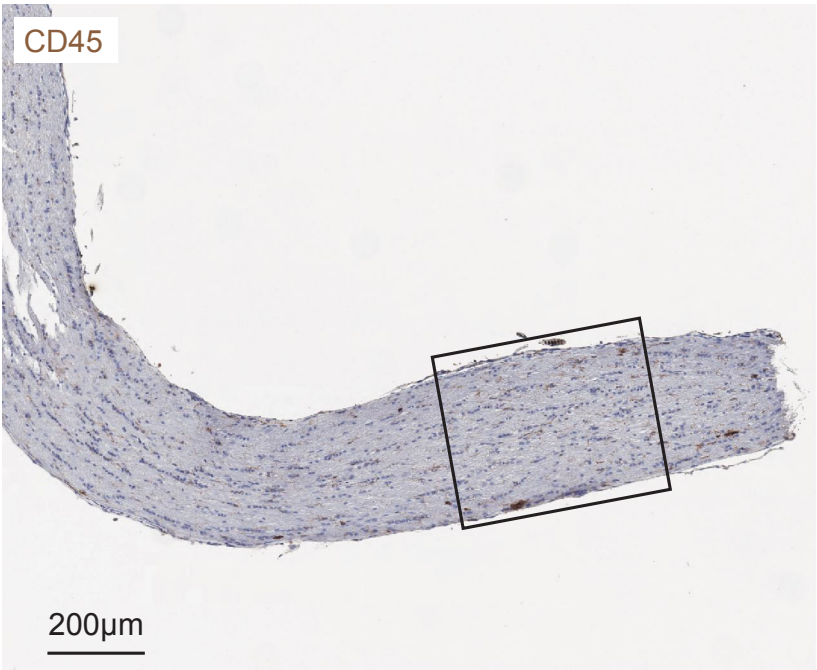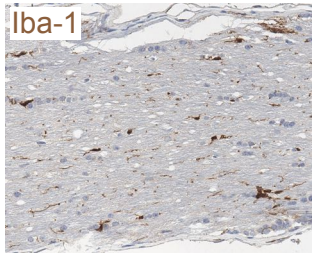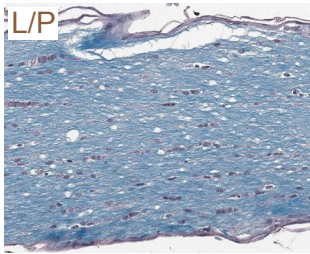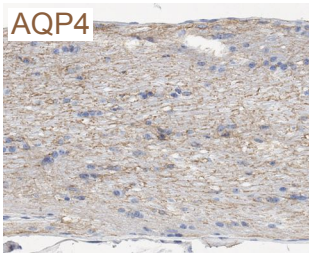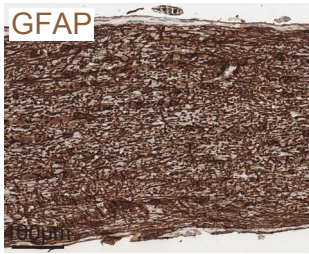

**a**

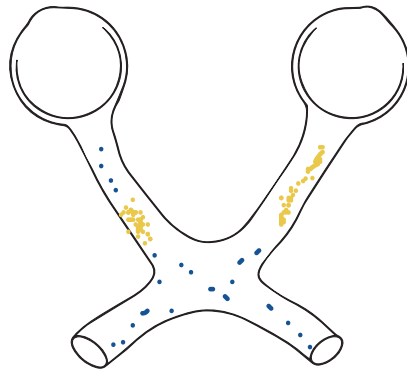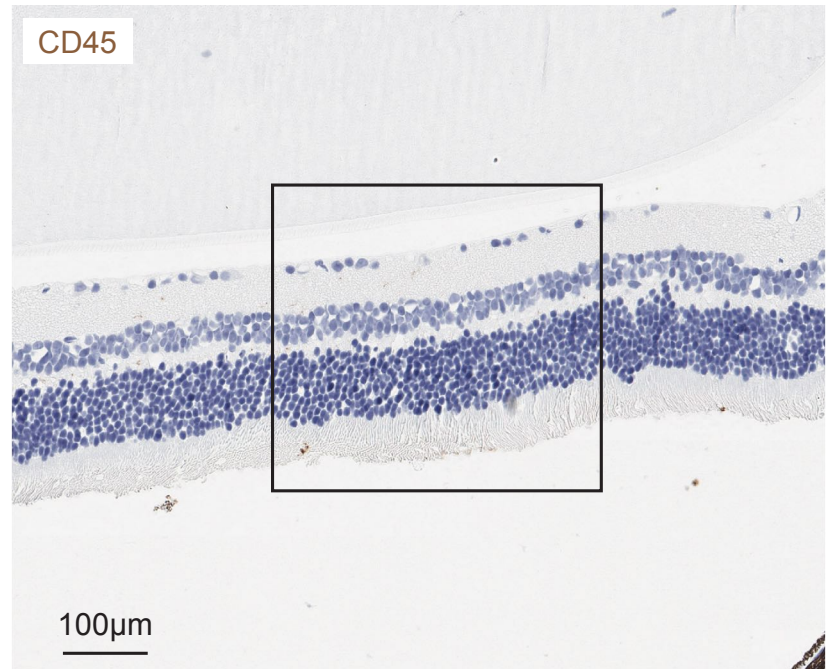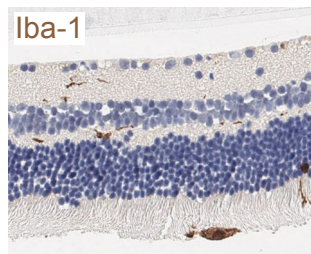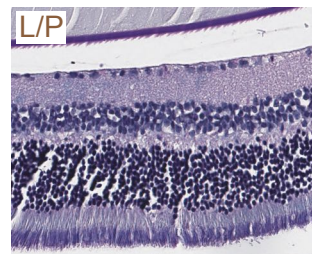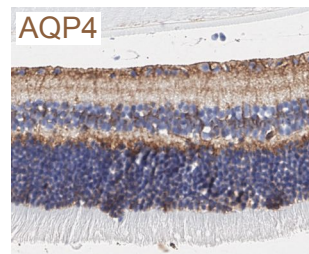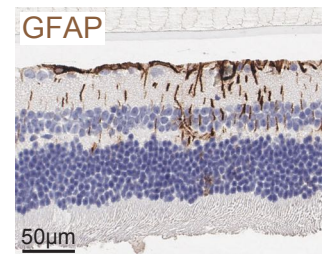

**b**

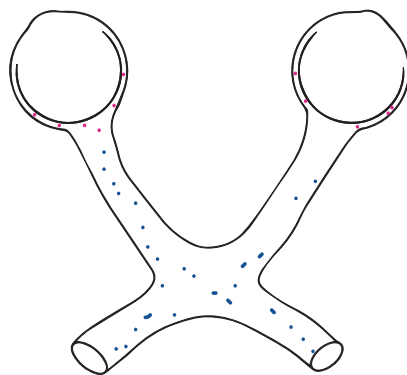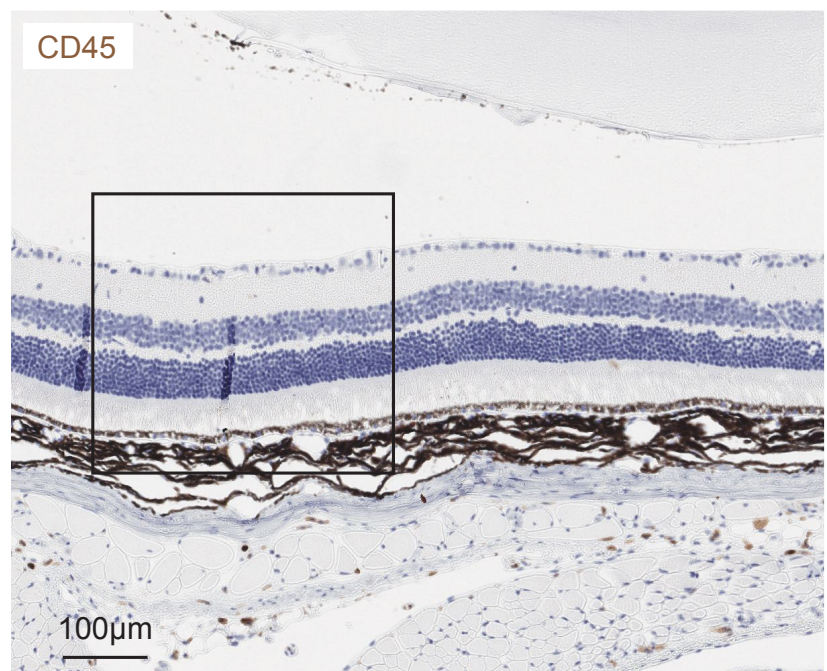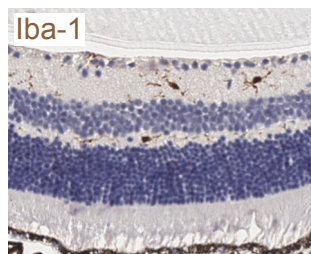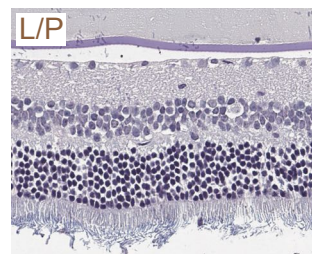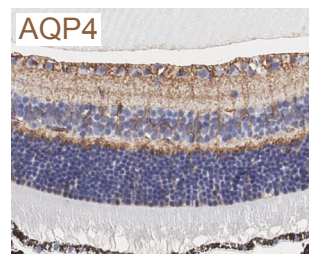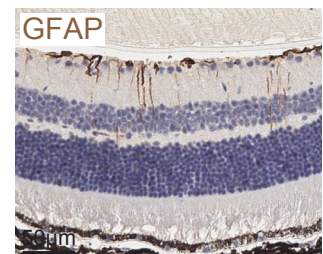

Supplement: Supplementary file 1 — Additional file 1. [file 40478_2025_1947_MOESM1_ESM.pdf]
