# Supplementary material for: AQP4-specific T cells determine lesion localization in the CNS in a model of NMOSD
Source: Acta Neuropathol Commun. 2025 Feb 11;13:27. doi: 10.1186/s40478-025-01947-8 (PMC11817536; doi:10.1186/s40478-025-01947-8)
Supplement: Supplementary file 2 — Additional file 2. [file 40478_2025_1947_MOESM2_ESM.docx]

**Legends to Supplementary Figures**

**Supplementary Figure 1. Flow cytometric characterization of mononuclear cell infiltrates of MOG(35-55)- vs. AQP4(201-220)-induced EAE.** (**a**) Schematic visualization of the experimental setup. Flow cytometric assessment of mononuclear cell infiltrates and intracellular cytokine staining of CD4^+^ T cells isolated from the CNS of either MOG(35-55)- or AQP4(201-220)-immunized *Aqp4*^ΔB^ mice at the peak of EAE (d15) and during recovery (d32). (**b**) Representative cytograms and gating strategy of mononuclear cell infiltrates. (**c**, **d**) Composition of CD45^+^ cells isolated from brain and spinal cord (SC) at different time points as indicated in the figure. (**e**) Representative cytograms of intracellular cytokine staining of CD4^+^Foxp3^–^ conventional T cells and CD4^+^Foxp3^+^ regulatory T (Treg) cells. (**f**) Fraction of CD4^+^Foxp3^+^ Treg cells and IL-10 expressors in the CNS CD4^+^Foxp3^+^ Treg cell compartment. (**g**) Fraction of cytokine expressors in the CNS conventional CD4^+^Foxp3^–^ T cell compartment. Data are shown as mean ± SD, n = 3 biological replicates.

**Supplementary Figure 2. Affection of peripheral organs in AQP4(201-220)-induced EAE.** Representative immunostaining for CD45 and AQP4 in kidney sections obtained from (**a**) MOG(35-55)- and (**b**) AQP4(201-220)-immunized *Aqp4*^ΔB^ mice at the peak of EAE. Scale bars (**a**, **b**) 500 µm (left) and 100 µm (top and bottom).

**Supplementary Figure 3. Resolution of inflammation in the CNS of mice with MOG(35-55)- vs. AQP4(201-220)-induced EAE.** Semiquantitative analysis of the lesion distribution in the CNS during recovery (d17 after disease onset) of (**a**) MOG(35-55)- and (**b**) AQP4(201-220)-induced EAE. Schemes were prepared according to a mouse CNS atlas [22]. Each data point represents a formation containing at least one to five CD45-immunoreactive cells as identified by QuPath automated cell detection. The coloring indicates whether the inflammatory infiltrates were present in a single group or in both, as specified in the legend.

**Supplementary Figure 4. Spinal cord pathology in MOG(35-55)- vs. AQP4(201-220)-induced EAE during recovery.** Representative immunostaining for CD45, Iba-1, LFB-PAS (L/P), AQP4, and GFAP in spinal cord sections obtained from (**a**) MOG(35-55)- and (**b**) AQP4(201-220)-immunized *Aqp4*^ΔB^ mice during recovery from EAE (n = 3 independent experiments). Scale bars (**a**, **b**) 400 µm (top) and 25 µm in enlarged image sections.

**Supplementary Figure 5. Topology and pathology of brain lesions in MOG(35-55)- vs. AQP4(201-220)-induced EAE during recovery.** Representative immunostaining for CD45, Iba-1, LFB-PAS (L/P), AQP4, and GFAP in the brains (sagittal section) of *Aqp4*^ΔB^ mice with (**a**) MOG(35-55)-induced and (**b**) AQP4(201-220)-induced EAE during recovery (n = 3 independent experiments). Scale bars 400 µm (top) and 100 µm in enlarged image sections.

**Supplementary Figure 6. Pathology of optic nerve lesions in MOG(35-55)- vs. AQP4(201-220)-induced EAE during recovery.** Representative immunostaining for CD45, Iba-1, LFB-PAS (L/P), AQP4, and GFAP in the optic nerves of *Aqp4*^ΔB^ mice with (**a**) MOG(35-55)-induced and (**b**) AQP4(201-220)-induced EAE during recovery (n = 3 independent experiments). Scale bars 200 µm (top) and 100 µm in enlarged image sections.

**Supplementary Figure 7. Retinal pathology in MOG(35-55)- vs. AQP4(201-220)-induced EAE during recovery.** Representative immunostaining for CD45, Iba-1, LFB-PAS (L/P), AQP4, and GFAP in the retinas of *Aqp4*^ΔB^ mice with (**a**) MOG(35-55)-induced and (**b**) AQP4(201-220)-induced EAE during recovery (n = 3 independent experiments). Scale bars 100 µm (top) and 50 µm in marked image sections.

**Supplementary material**

**Antibodies for flow cytometry**

| Marker | Clone | Species | Fluoro-phore | Company | Catalog# | Titration | RRID |
| --- | --- | --- | --- | --- | --- | --- | --- |
| CD3e | SK7 | Mouse | PE-Cy7 | BD | 341111 | 1:300 | AB_10596664 |
| CD4 | GK1.5 | Rat | BV421 | BioLegend | 100438 | 1:500 | AB_2562557 |
| CD8a | 53-6.7 | Rat | BV510 | BioLegend | 100752 | 1:300 | AB_2563057 |
| CD11b | M1/70 | Rat | BV510 | BioLegend | 101263 | 1:300 | AB_2629529 |
| CD11b | M1/70 | Rat | FITC | BioLegend | 101205 | 1:300 | AB_312788 |
| CD16/ CD32 | 2.4G2 | Rat | - | BD | 553142 | 1:100 | AB_394657 |
| CD19 | 1D3 | Rat | PE | BD | 557399 | 1:300 | AB_395050 |
| CD19 | 6D5 | Rat | BV510 | BioLegend | 115546 | 1:300 | AB_2562136 |
| CD45 | 30-F11 | Rat | PerCP-Cy5.5 | BioLegend | 103132 | 1:300 | AB_893340 |
| F4/80 | BM8 | Rat | BV510 | BioLegend | 123135 | 1:300 | AB_2562622 |
| Foxp3 | FJK-16s | Rat | AF488 | eBioscience | 53-5773 | 1:200 | AB_763537 |
| GM-CSF | MP1-22E9 | Rat | PE | BD | 554406 | 1:200 | AB_395371 |
| IFN-y | XMG1.2 | Rat | APC | Biolegend | 652408 | 1:200 | AB_2562139 |
| IL-10 | JES5-16E3 | Rat | PE-Cy7 | BioLegend | 505026 | 1:200 | AB_315361 |
| IL-17a | TC11-18H10 | Rat | PerCP-Cy5.5 | BD | 560666 | 1:200 | AB_1937311 |
| NK1.1 | PK136 | Mouse | BV510 | BioLegend | 108738 | 1:300 | AB_2562216 |

**Antibodies for immunohistochemistry**

| Marker | Clone | Species | Company | Catalog# | Titration | Validation |
| --- | --- | --- | --- | --- | --- | --- |
| AQP4 | Polyclonal | Rat | Sigma | HPA014784 | 1:2000 | Manufacturer |
| CD45 | 30-F11 | Rat | Thermo | 14-0451-82 | 1:500 | AB_467251 |
| GFAP | G-A-5 | Mouse | Sigma | G6171 | 1:400 | Manufacturer |
| Iba1 | Polyclonal | Rabbit | Wako | 019–19,741 | 1:500 | Manufacturer |
